# *catena*-Poly[[tetra­aqua­nickel(II)]-μ-(9,10-dioxo-9,10-dihydro­anthracene-1,4,5,8-tetra­carboxyl­ato)-κ^2^ *O* ^1^:*O* ^8^-[tetra­aqua­nickel(II)]-μ-4,4′-bipyridine-κ^2^ *N*:*N*′]

**DOI:** 10.1107/S1600536812027493

**Published:** 2012-06-23

**Authors:** Yang-Mei Liu, Kai Cao, Feng-Lin Wang

**Affiliations:** a National Food Packaging Products Quality Supervision and Inspection Center, Jiangsu Provincial Supervising and Testing Research Institute for Products Quality, Nanjing 210007, People’s Republic of China; b School of Chemistry and Chemical Engineering, Nanjing University, Nanjing 210093, People’s Republic of China

## Abstract

In the crystal of the title polymeric complex, [Ni_2_(C_18_H_4_O_10_)(C_10_H_8_N_2_)(H_2_O)_8_]_*n*_, each Ni^II^ cation is coord­in­ated by four water mol­ecules in the equatorial plane, and is further bridged by an 9,10-dioxo-9,10-dihydro­anthracene-1,4,5,8-tetra­carb­oxy­l­ate anion and a 4,4′-bipyridine ligand in the axial directions, forming a distorted octa­hedral geometry. The 9,10-dioxo-9,10-dihydro­anthracene-1,4,5,8-tetra­carboxyl­ate anion is centrosymmetric with the centroid of the quinone ring located about an inversion center; the 4,4′-bipyridine ligand is also centrosymmetric with the mid-point of the C—C bond linking two pyridine rings located about another invertion center. The 9,10-dioxo-9,10-dihydro­anthracene-1,4,5,8-tetra­carboxyl­ate anion and bypiridine ligand bridge the Ni^II^ cations, forming a polymeric chain along the *b* axis. π–π stacking is observed between pyridine and benzene rings [centroid–centroid distance = 3.705 (2) Å]. All of the coordinating water mol­ecules are involved in O—H⋯O hydrogen bonding.

## Related literature

For the synthesis, see: Liu *et al.* (2010 ▶).
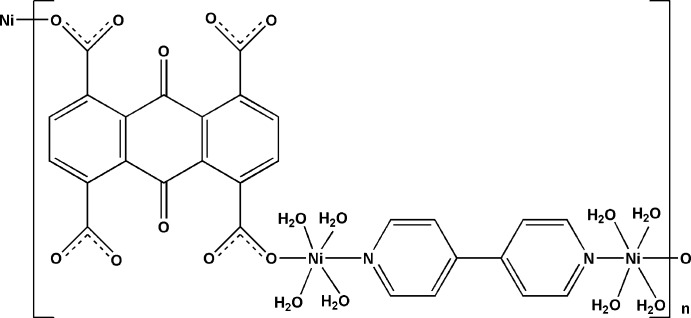


## Experimental

### 

#### Crystal data

[Ni_2_(C_18_H_4_O_10_)(C_10_H_8_N_2_)(H_2_O)_8_]
*M* *_r_* = 797.94
Monoclinic, 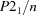

*a* = 8.6359 (16) Å
*b* = 21.2426 (12) Å
*c* = 8.6416 (13) Å
β = 92.789 (3)°
*V* = 1583.4 (4) Å^3^
*Z* = 2
Mo *K*α radiation
μ = 1.28 mm^−1^
*T* = 291 K
0.28 × 0.24 × 0.22 mm

#### Data collection

Bruker SMART APEXII CCD diffractometer
Absorption correction: multi-scan (*SADABS*; Bruker, 2001 ▶) *T*_min_ = 0.716, *T*_max_ = 0.767
8595 measured reflections
3111 independent reflections
2297 reflections with *I* > 2σ(*I*)
*R*_int_ = 0.056

#### Refinement

*R*[*F* ^2^ > 2σ(*F* ^2^)] = 0.048
*wR*(*F* ^2^) = 0.102
*S* = 1.00
3111 reflections
226 parameters
H-atom parameters constrained
Δρ_max_ = 0.86 e Å^−3^
Δρ_min_ = −0.49 e Å^−3^

### 

Data collection: *APEX2* (Bruker, 2007 ▶); cell refinement: *SAINT* (Bruker, 2007 ▶); data reduction: *SAINT*; program(s) used to solve structure: *SHELXTL* (Sheldrick, 2008 ▶); program(s) used to refine structure: *SHELXTL*; molecular graphics: *SHELXTL*; software used to prepare material for publication: *SHELXTL*.

## Supplementary Material

Crystal structure: contains datablock(s) I, global. DOI: 10.1107/S1600536812027493/xu5560sup1.cif

Structure factors: contains datablock(s) I. DOI: 10.1107/S1600536812027493/xu5560Isup2.hkl

Additional supplementary materials:  crystallographic information; 3D view; checkCIF report

## Figures and Tables

**Table 1:** Hydrogen-bond geometry (Å, °)

*D*—H⋯*A*	*D*—H	H⋯*A*	*D*⋯*A*	*D*—H⋯*A*
O6—H6*X*⋯O4	0.85	2.14	2.642 (4)	118
O6—H6*Y*⋯O1^i^	0.85	2.16	2.630 (3)	115
O7—H7*Y*⋯O2^i^	0.85	1.82	2.618 (3)	155
O7—H7*X*⋯O4^ii^	0.85	1.88	2.717 (4)	168
O8—H8*X*⋯O6^ii^	0.85	2.42	3.243 (4)	164
O8—H8*Y*⋯O7^iii^	0.85	2.50	3.330 (4)	167
O9—H9*X*⋯O7^iii^	0.85	2.17	2.973 (3)	158
O9—H9*Y*⋯O3	0.85	2.26	3.099 (3)	170

Symmetry codes: (i) 
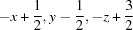
; (ii) 
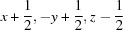
; (iii) 
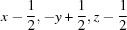
.

**Fig. 1.**
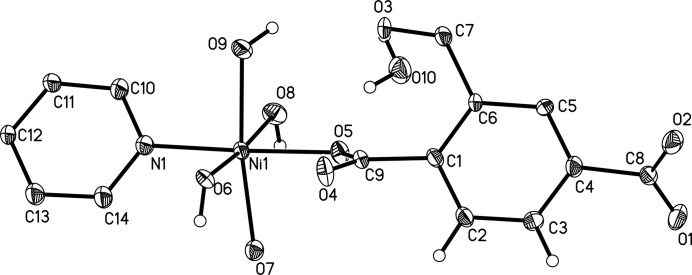
The molecular structure of the title compound. Displacement ellipsoids are drawn at the 50% probability level.

**Fig. 2.**
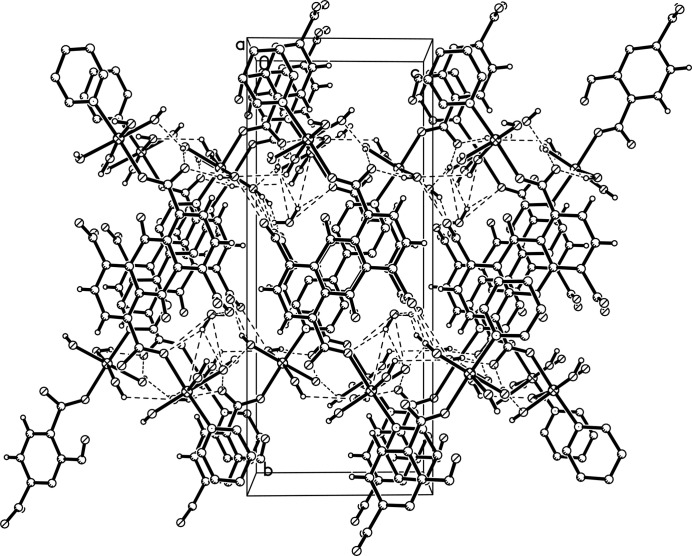
The packing of (I), viewed down the a axis.

## supplementary crystallographic information

### Comment

The molecular structure of the title complex (I) is showing in Fig. 1. The asymmetric unit of I contains one independent nickel(II) ion, a half 9,10-dioxo-9,10-dihydroanthracene-1,4,5,8-tetracarboxylic acid ligand, a half 4,4-bipyridine ligand, four coordinating water molecules.

The nickel atom is in a normal octahedral geometry with O atoms which from the coordinating water molecules on the equatorial plane and O atom from the carboxylate group, N atom from the 4,4-bipyridine in the axial position. Every nickel atom is coordinated with one 9,10-dioxo-9,10-dihydroanthracene-1,4,5,8-tetracarboxylate ligand and one 4,4-bipyridine ligand. And every 9,10-dioxo-9,10-dihydroanthracene-1,4,5,8-tetracarboxylic acid ligand coordinates with two nickel atoms and they form a one-dimensional nickel chain along *b* axis. There are two nickel chains which cross each other in the crystal structure.

### Experimental

The title compound was obtained unintentionally as the product of an attempted synthesis of a polymeric network Ni complex with 9,10-dioxo-9,10-dihydroanthracene-1,4,5,8-tetracarboxylic acid and 4,4-bipyridine (Liu *et al.*, 2010), the pH value of the mixture was adjusted to 8.0 with NaOH solution, and then solution was heated at 393 K for 3 d. After the mixture was slowly cooled to room temperature, green crystals of the title compound were obtained.

### Refinement

Water H atoms were located in a difference map and refined with distance restraints of O—H = 0.85 Å, other H atoms were placed in calculated positions with C—H = 0.93 Å and refined in riding mode. *U*_iso_(H) = 1.2*U*_eq_(C,O).

### Crystal data

**Table:**

[Ni_2_(C_18_H_4_O_10_)(C_10_H_8_N_2_)(H_2_O)_8_]	*F*(000) = 820
*M**_r_* = 797.94	*D*_x_ = 1.674 Mg m^−^^3^
Monoclinic, *P*2_1_/*n*	Mo *K*α radiation, λ = 0.71073 Å
Hall symbol: -P 2yn	Cell parameters from 1207 reflections
*a* = 8.6359 (16) Å	θ = 2.5–21.8°
*b* = 21.2426 (12) Å	µ = 1.28 mm^−^^1^
*c* = 8.6416 (13) Å	*T* = 291 K
β = 92.789 (3)°	Block, green
*V* = 1583.4 (4) Å^3^	0.28 × 0.24 × 0.22 mm
*Z* = 2	

### Data collection

**Table:**

Bruker SMART APEXII CCD diffractometer	3111 independent reflections
Radiation source: sealed tube	2297 reflections with *I* > 2σ(*I*)
Graphite monochromator	*R*_int_ = 0.056
φ and ω scans	θ_max_ = 26.0°, θ_min_ = 1.9°
Absorption correction: multi-scan (*SADABS*; Bruker, 2001)	*h* = −9→10
*T*_min_ = 0.716, *T*_max_ = 0.767	*k* = −26→25
8595 measured reflections	*l* = −7→10

### Refinement

**Table:**

Refinement on *F*^2^	Primary atom site location: structure-invariant direct methods
Least-squares matrix: full	Secondary atom site location: difference Fourier map
*R*[*F*^2^ > 2σ(*F*^2^)] = 0.048	Hydrogen site location: inferred from neighbouring sites
*wR*(*F*^2^) = 0.102	H-atom parameters constrained
*S* = 1.00	*w* = 1/[σ^2^(*F*_o_^2^) + (0.04*P*)^2^ + 1.66*P*] where *P* = (*F*_o_^2^ + 2*F*_c_^2^)/3
3111 reflections	(Δ/σ)_max_ < 0.001
226 parameters	Δρ_max_ = 0.86 e Å^−^^3^
0 restraints	Δρ_min_ = −0.49 e Å^−^^3^

### Special details

**Table:**

Geometry. All e.s.d.'s (except the e.s.d. in the dihedral angle between two l.s. planes) are estimated using the full covariance matrix. The cell e.s.d.'s are taken into account individually in the estimation of e.s.d.'s in distances, angles and torsion angles; correlations between e.s.d.'s in cell parameters are only used when they are defined by crystal symmetry. An approximate (isotropic) treatment of cell e.s.d.'s is used for estimating e.s.d.'s involving l.s. planes.
Refinement. Refinement of *F*^2^ against ALL reflections. The weighted *R*-factor *wR* and goodness of fit *S* are based on *F*^2^, conventional *R*-factors *R* are based on *F*, with *F* set to zero for negative *F*^2^. The threshold expression of *F*^2^ > σ(*F*^2^) is used only for calculating *R*-factors(gt) *etc*. and is not relevant to the choice of reflections for refinement. *R*-factors based on *F*^2^ are statistically about twice as large as those based on *F*, and *R*- factors based on ALL data will be even larger.

### Fractional atomic coordinates and isotropic or equivalent isotropic displacement parameters (Å^2^)

**Table:**

	*x*	*y*	*z*	*U*_iso_*/*U*_eq_	
C1	0.1039 (5)	0.38729 (17)	0.6495 (5)	0.0279 (9)	
C2	0.2016 (4)	0.39108 (17)	0.7843 (4)	0.0231 (8)	
H2	0.2351	0.3543	0.8334	0.028*	
C3	0.2482 (4)	0.44891 (17)	0.8487 (4)	0.0219 (8)	
H3	0.3128	0.4499	0.9380	0.026*	
C4	0.1983 (5)	0.50468 (18)	0.7798 (4)	0.0253 (8)	
C5	0.1014 (4)	0.50163 (17)	0.6407 (4)	0.0197 (7)	
C6	0.0513 (4)	0.44257 (18)	0.5757 (4)	0.0243 (8)	
C7	−0.0636 (4)	0.43979 (16)	0.4486 (4)	0.0177 (7)	
C8	0.2460 (5)	0.56481 (18)	0.8562 (4)	0.0254 (8)	
C9	0.0736 (4)	0.32135 (17)	0.5866 (4)	0.0225 (8)	
C10	0.0110 (4)	0.14286 (18)	0.0564 (4)	0.0228 (8)	
H10	−0.0205	0.1805	0.0100	0.027*	
C11	−0.0207 (4)	0.08759 (16)	−0.0196 (4)	0.0222 (8)	
H11	−0.0731	0.0895	−0.1161	0.027*	
C12	0.0200 (4)	0.02965 (17)	0.0378 (4)	0.0223 (8)	
C13	0.1022 (4)	0.03075 (17)	0.1803 (4)	0.0226 (8)	
H13	0.1327	−0.0067	0.2285	0.027*	
C14	0.1388 (5)	0.08895 (17)	0.2511 (4)	0.0265 (8)	
H14	0.2024	0.0894	0.3411	0.032*	
N1	0.0841 (3)	0.14441 (14)	0.1922 (4)	0.0229 (7)	
Ni1	0.10940 (5)	0.22477 (2)	0.33004 (5)	0.02030 (14)	
O1	0.3792 (3)	0.57569 (12)	0.8713 (3)	0.0307 (7)	
O2	0.1359 (3)	0.59770 (12)	0.9128 (3)	0.0290 (6)	
O3	−0.1319 (3)	0.39084 (11)	0.4195 (3)	0.0222 (6)	
O4	0.0032 (3)	0.28515 (12)	0.6693 (3)	0.0293 (6)	
O5	0.1290 (3)	0.30879 (11)	0.4574 (3)	0.0242 (6)	
O6	0.0040 (3)	0.17982 (11)	0.5068 (3)	0.0214 (5)	
H6X	0.0512	0.1923	0.5898	0.026*	
H6Y	−0.0052	0.1415	0.4795	0.026*	
O7	0.3310 (3)	0.20054 (11)	0.4219 (3)	0.0204 (5)	
H7X	0.3965	0.2055	0.3525	0.024*	
H7Y	0.3314	0.1623	0.4510	0.024*	
O8	0.1681 (3)	0.27455 (12)	0.1160 (3)	0.0289 (6)	
H8X	0.2466	0.2873	0.0692	0.035*	
H8Y	0.0907	0.2797	0.0526	0.035*	
O9	−0.1130 (3)	0.26235 (12)	0.2516 (3)	0.0269 (6)	
H9X	−0.1200	0.2630	0.1531	0.032*	
H9Y	−0.1223	0.2996	0.2861	0.032*	

### Atomic displacement parameters (Å^2^)

**Table:**

	*U*^11^	*U*^22^	*U*^33^	*U*^12^	*U*^13^	*U*^23^
C1	0.038 (2)	0.0167 (19)	0.029 (2)	−0.0067 (17)	0.0048 (17)	0.0011 (16)
C2	0.030 (2)	0.0183 (18)	0.0221 (19)	0.0004 (15)	0.0091 (15)	0.0003 (14)
C3	0.0259 (19)	0.0222 (19)	0.0173 (18)	−0.0010 (15)	−0.0017 (14)	−0.0068 (14)
C4	0.032 (2)	0.0183 (18)	0.026 (2)	−0.0035 (16)	0.0039 (16)	−0.0004 (15)
C5	0.0205 (18)	0.0158 (17)	0.0234 (19)	0.0034 (14)	0.0068 (14)	−0.0014 (14)
C6	0.0250 (19)	0.028 (2)	0.0190 (19)	0.0009 (16)	−0.0060 (15)	−0.0030 (15)
C7	0.0182 (16)	0.0215 (19)	0.0129 (17)	−0.0045 (14)	−0.0039 (13)	−0.0033 (13)
C8	0.032 (2)	0.027 (2)	0.0173 (18)	−0.0026 (17)	−0.0018 (15)	−0.0035 (15)
C9	0.0210 (18)	0.0227 (19)	0.025 (2)	0.0110 (15)	0.0092 (15)	0.0008 (15)
C10	0.034 (2)	0.0190 (18)	0.0156 (18)	0.0016 (16)	0.0027 (15)	−0.0020 (14)
C11	0.034 (2)	0.0181 (18)	0.0145 (18)	−0.0110 (16)	−0.0041 (14)	−0.0021 (14)
C12	0.0266 (19)	0.0186 (19)	0.0213 (19)	−0.0064 (15)	−0.0029 (14)	−0.0054 (15)
C13	0.0229 (19)	0.0194 (18)	0.026 (2)	0.0073 (15)	0.0082 (15)	0.0004 (15)
C14	0.034 (2)	0.0195 (19)	0.025 (2)	−0.0061 (16)	−0.0079 (16)	0.0010 (15)
N1	0.0224 (15)	0.0182 (15)	0.0271 (17)	0.0018 (13)	−0.0096 (13)	−0.0073 (13)
Ni1	0.0171 (2)	0.0217 (2)	0.0218 (2)	0.0006 (2)	−0.00221 (16)	−0.0025 (2)
O1	0.0302 (16)	0.0160 (13)	0.0459 (18)	−0.0110 (12)	0.0011 (13)	−0.0077 (12)
O2	0.0299 (15)	0.0217 (14)	0.0342 (16)	0.0048 (11)	−0.0101 (12)	−0.0123 (11)
O3	0.0311 (14)	0.0136 (13)	0.0200 (13)	0.0032 (10)	−0.0177 (11)	−0.0030 (10)
O4	0.0465 (17)	0.0224 (14)	0.0193 (13)	−0.0118 (12)	0.0035 (12)	−0.0132 (11)
O5	0.0356 (15)	0.0155 (13)	0.0219 (13)	0.0004 (11)	0.0049 (11)	0.0015 (10)
O6	0.0235 (13)	0.0210 (13)	0.0193 (13)	0.0063 (11)	−0.0032 (10)	0.0017 (10)
O7	0.0194 (12)	0.0205 (13)	0.0206 (13)	−0.0057 (10)	−0.0064 (10)	0.0019 (10)
O8	0.0405 (16)	0.0186 (13)	0.0279 (15)	−0.0069 (12)	0.0047 (12)	0.0000 (11)
O9	0.0299 (14)	0.0215 (14)	0.0286 (14)	0.0075 (11)	−0.0054 (11)	0.0006 (11)

### Geometric parameters (Å, º)

**Table:**

C1—C6	1.401 (5)	C11—H11	0.9300
C1—C2	1.407 (6)	C12—C13	1.391 (5)
C1—C9	1.521 (5)	C12—C12^ii^	1.453 (7)
C2—C3	1.400 (5)	C13—C14	1.409 (5)
C2—H2	0.9298	C13—H13	0.9300
C3—C4	1.385 (5)	C14—N1	1.359 (5)
C3—H3	0.9300	C14—H14	0.9300
C4—C5	1.432 (5)	N1—Ni1	2.087 (3)
C4—C8	1.487 (5)	Ni1—O6	2.051 (2)
C5—C6	1.433 (5)	Ni1—O5	2.099 (3)
C5—C7^i^	1.492 (5)	Ni1—O7	2.100 (2)
C6—C7	1.445 (5)	Ni1—O9	2.159 (3)
C7—O3	1.215 (4)	Ni1—O8	2.211 (3)
C7—C5^i^	1.492 (5)	O6—H6X	0.8500
C8—O1	1.174 (5)	O6—H6Y	0.8500
C8—O2	1.295 (5)	O7—H7X	0.8500
C9—O4	1.230 (4)	O7—H7Y	0.8500
C9—O5	1.265 (4)	O8—H8X	0.8505
C10—N1	1.305 (5)	O8—H8Y	0.8498
C10—C11	1.366 (5)	O9—H9X	0.8500
C10—H10	0.9300	O9—H9Y	0.8499
C11—C12	1.367 (5)		
			
C6—C1—C2	119.8 (3)	C12—C13—H13	120.2
C6—C1—C9	124.3 (4)	C14—C13—H13	120.2
C2—C1—C9	115.7 (3)	N1—C14—C13	122.1 (3)
C3—C2—C1	121.9 (3)	N1—C14—H14	119.0
C3—C2—H2	118.6	C13—C14—H14	119.0
C1—C2—H2	119.4	C10—N1—C14	117.3 (3)
C4—C3—C2	120.1 (3)	C10—N1—Ni1	124.5 (3)
C4—C3—H3	119.9	C14—N1—Ni1	118.0 (2)
C2—C3—H3	119.9	O6—Ni1—N1	90.37 (12)
C3—C4—C5	118.6 (3)	O6—Ni1—O5	91.88 (10)
C3—C4—C8	118.0 (3)	N1—Ni1—O5	176.51 (11)
C5—C4—C8	123.3 (3)	O6—Ni1—O7	91.93 (10)
C4—C5—C6	121.4 (3)	N1—Ni1—O7	94.80 (10)
C4—C5—C7^i^	120.0 (3)	O5—Ni1—O7	87.80 (10)
C6—C5—C7^i^	118.3 (3)	O6—Ni1—O9	89.18 (10)
C1—C6—C5	118.1 (3)	N1—Ni1—O9	93.22 (11)
C1—C6—C7	120.5 (3)	O5—Ni1—O9	84.14 (10)
C5—C6—C7	121.1 (3)	O7—Ni1—O9	171.90 (10)
O3—C7—C6	120.0 (3)	O6—Ni1—O8	166.82 (10)
O3—C7—C5^i^	121.0 (3)	N1—Ni1—O8	86.30 (11)
C6—C7—C5^i^	118.9 (3)	O5—Ni1—O8	90.92 (9)
O1—C8—O2	125.8 (4)	O7—Ni1—O8	101.05 (10)
O1—C8—C4	117.9 (4)	O9—Ni1—O8	78.29 (11)
O2—C8—C4	116.0 (3)	C9—O5—Ni1	128.2 (2)
O4—C9—O5	126.9 (4)	Ni1—O6—H6X	105.7
O4—C9—C1	116.7 (3)	Ni1—O6—H6Y	106.1
O5—C9—C1	116.3 (3)	H6X—O6—H6Y	124.6
N1—C10—C11	122.0 (4)	Ni1—O7—H7X	109.2
N1—C10—H10	119.0	Ni1—O7—H7Y	109.6
C11—C10—H10	119.0	H7X—O7—H7Y	109.5
C10—C11—C12	123.9 (3)	Ni1—O8—H8X	140.4
C10—C11—H11	118.1	Ni1—O8—H8Y	113.3
C12—C11—H11	118.1	H8X—O8—H8Y	105.8
C11—C12—C13	114.7 (3)	Ni1—O9—H9X	109.8
C11—C12—C12^ii^	124.5 (4)	Ni1—O9—H9Y	109.3
C13—C12—C12^ii^	120.8 (4)	H9X—O9—H9Y	109.5
C12—C13—C14	119.6 (3)		
			
C6—C1—C2—C3	0.3 (6)	C2—C1—C9—O4	64.4 (5)
C9—C1—C2—C3	175.5 (3)	C6—C1—C9—O5	62.1 (5)
C1—C2—C3—C4	0.0 (6)	C2—C1—C9—O5	−112.8 (4)
C2—C3—C4—C5	−1.2 (6)	N1—C10—C11—C12	0.1 (6)
C2—C3—C4—C8	177.6 (4)	C10—C11—C12—C13	−1.9 (6)
C3—C4—C5—C6	2.3 (5)	C10—C11—C12—C12^ii^	176.5 (5)
C8—C4—C5—C6	−176.5 (4)	C11—C12—C13—C14	−1.2 (5)
C3—C4—C5—C7^i^	−171.5 (3)	C12^ii^—C12—C13—C14	−179.7 (4)
C8—C4—C5—C7^i^	9.7 (6)	C12—C13—C14—N1	6.3 (6)
C2—C1—C6—C5	0.7 (6)	C11—C10—N1—C14	4.9 (6)
C9—C1—C6—C5	−174.0 (3)	C11—C10—N1—Ni1	−170.8 (3)
C2—C1—C6—C7	−172.5 (3)	C13—C14—N1—C10	−8.1 (6)
C9—C1—C6—C7	12.8 (6)	C13—C14—N1—Ni1	167.9 (3)
C4—C5—C6—C1	−2.0 (6)	C10—N1—Ni1—O6	122.4 (3)
C7^i^—C5—C6—C1	171.9 (3)	C14—N1—Ni1—O6	−53.4 (3)
C4—C5—C6—C7	171.1 (3)	C10—N1—Ni1—O7	−145.7 (3)
C7^i^—C5—C6—C7	−15.0 (6)	C14—N1—Ni1—O7	38.6 (3)
C1—C6—C7—O3	10.8 (6)	C10—N1—Ni1—O9	33.2 (3)
C5—C6—C7—O3	−162.2 (3)	C14—N1—Ni1—O9	−142.6 (3)
C1—C6—C7—C5^i^	−171.9 (3)	C10—N1—Ni1—O8	−44.9 (3)
C5—C6—C7—C5^i^	15.1 (6)	C14—N1—Ni1—O8	139.4 (3)
C3—C4—C8—O1	60.7 (5)	O4—C9—O5—Ni1	4.4 (6)
C5—C4—C8—O1	−120.5 (4)	C1—C9—O5—Ni1	−178.6 (2)
C3—C4—C8—O2	−113.1 (4)	O6—Ni1—O5—C9	−6.8 (3)
C5—C4—C8—O2	65.7 (5)	O9—Ni1—O5—C9	82.2 (3)
C6—C1—C9—O4	−120.6 (4)	O8—Ni1—O5—C9	160.3 (3)

Symmetry codes: (i) −*x*, −*y*+1, −*z*+1; (ii) −*x*, −*y*, −*z*.

### Hydrogen-bond geometry (Å, º)

**Table:**

*D*—H···*A*	*D*—H	H···*A*	*D*···*A*	*D*—H···*A*
O6—H6*X*···O4	0.85	2.14	2.642 (4)	118
O6—H6*Y*···O1^iii^	0.85	2.16	2.630 (3)	115
O7—H7*Y*···O2^iii^	0.85	1.82	2.618 (3)	155
O7—H7*X*···O4^iv^	0.85	1.88	2.717 (4)	168
O8—H8*X*···O6^iv^	0.85	2.42	3.243 (4)	164
O8—H8*Y*···O7^v^	0.85	2.50	3.330 (4)	167
O9—H9*X*···O7^v^	0.85	2.17	2.973 (3)	158
O9—H9*Y*···O3	0.85	2.26	3.099 (3)	170

Symmetry codes: (iii) −*x*+1/2, *y*−1/2, −*z*+3/2; (iv) *x*+1/2, −*y*+1/2, *z*−1/2; (v) *x*−1/2, −*y*+1/2, *z*−1/2.
